# Antimicrobial and immunomodulatory responses of photodynamic therapy in *Galleria mellonella* model

**DOI:** 10.1186/s12866-020-01882-9

**Published:** 2020-07-06

**Authors:** Xiaowen Huang, Meinian Xu, Wen Pan, Menglei Wang, Xiaoyan Wu, Siqi Dai, Li Li, Kang Zeng

**Affiliations:** 1 Department of Dermatology, Nanfang Hospital, Southern Medical University, 1838 North Avenue, Guangzhou, 510515 China; 2 Division of Infectious Diseases, Rhode Island Hospital, Warren Alpert Medical School of Brown University, Providence, RI USA; 3 Department of Dermatology and Venereology, The Third Affiliated Hospital of Sun Yat-Sen University, Guangzhou, China

**Keywords:** Photodynamic therapy, *Galleria mellonella*, Hemocytes, *Fonsecaea monophora*, Innate immunity

## Abstract

**Background:**

New therapeutics are urgently needed for infectious diseases, especially for the fungal infection like *Fonsecaea monophora*. Photodynamic therapy has been showing antimicrobial activity on some pathogens. The combination of antimicrobial medicines and photodynamic therapy (PDT) might be a practical approach. However, whether the treatment of PDT could do benefits to the host immunity remains poorly documented.

**Results:**

In this study, *Galleria mellonella* larvae were employed as a model organism to evaluate the activity of PDT, and also to investigate the regulation of humoral immunity by PDT. Photosensitizer 5-aminolevulinic acid (ALA) was applied to the *G. mellonella* infection model. It was found that ALA-mediated PDT was non-toxic to *G. mellonella,* and could extend the median survival of infected larvae from 3 days to 5.5 days. We observed that larval hemocytes inhibited the growth of *Candida albicans* and *Staphylococcus aureus*, without any contribution by ALA-PDT. Furthermore, the application of ALA-PDT demonstrated the immunomodulation of larval innate immunity as increased hemocyte density counting, cell morphological transformation, and sensitivity to pathogens.

**Conclusions:**

*G. mellonella* could be considered as a useful model to study the immunoregulation of PDT. This model revealed that ALA-PDT positively defense against infections through inducing humoral immune responses of larvae.

## Background

Chromoblastomycosis (CBM) is a chronic skin disease caused by infection with dematiaceous fungi. Increasing refractory cases have been reported in the past few years [1]. Usually, treatment with antifungal drugs for CBM takes an extended period, and sometimes the efficacy is poor. There are other treatments for CBM, including surgical resection, cryotherapy with liquid nitrogen, carbon dioxide laser therapy, and hyperthermia therapy. These treatments can be applied alone or in combination, depending on the size and localization of lesions. But to date, there is no available standard therapy for CBM.

Recently, the application of antimicrobial photodynamic therapy (PDT) has proved to be efficient in relieving CBM lesions. We previously reported two cases of CBM cured by 5-aminolevulinic acid (ALA)-mediated PDT combined with antifungal agents [2, 3], and verified ALA-PDT had fungicidal effects on *Fonsecaea monophora* in vitro. Moreover, some researchers employed macrophages as a study model and found that ALA-PDT could enhance the fungicidal ability of macrophages stimulated by fungal conidia [4]. However, these results were limited to the in vitro systems. Developing simple animal models for exploring PDT-mediated immunomodulatory mechanisms is warranted.

Alternative invertebrate hosts, such as the greater wax moth *Galleria mellonella*, provide new possibilities for investigating microbial pathogenesis and evaluating novel therapies [5]. Lots of research revealed that *G. mellonella* is a prominent infection model organism to investigate various microbial pathogens [6, 7]. We have successfully established *G. mellonella* – dematiaceous fungi infection model and evaluated the virulence of different pathogenic strains [8]. It was well-known that the larvae of *G. mellonella* have an immune system composed of cellular and humoral responses similar to humans [9], therefore, *G. mellonella* could be used to evaluate the immunomodulatory potential in the host. Especially, larvae have been considered as excellent model animal for studying innate immunity against pathogenic microbial agents by looking into hemocytes development and differentiation [10].

The objectives of this study were to extend the *G. mellonella* infection model in exploring the antimicrobial effects of PDT and to identify the regulation of innate immunity by PDT in larvae. We observed that ALA-PDT prolonged the survival of *G. mellonella* against infection and induced the enhanced host innate immunity. The results here indicated that the *G. mellonella* infection model is novel and valuable for revealing the immune regulatory effect of PDT.

## Results

### Survival of G. mellonella infected with F. monophora

**Fig. 1:**
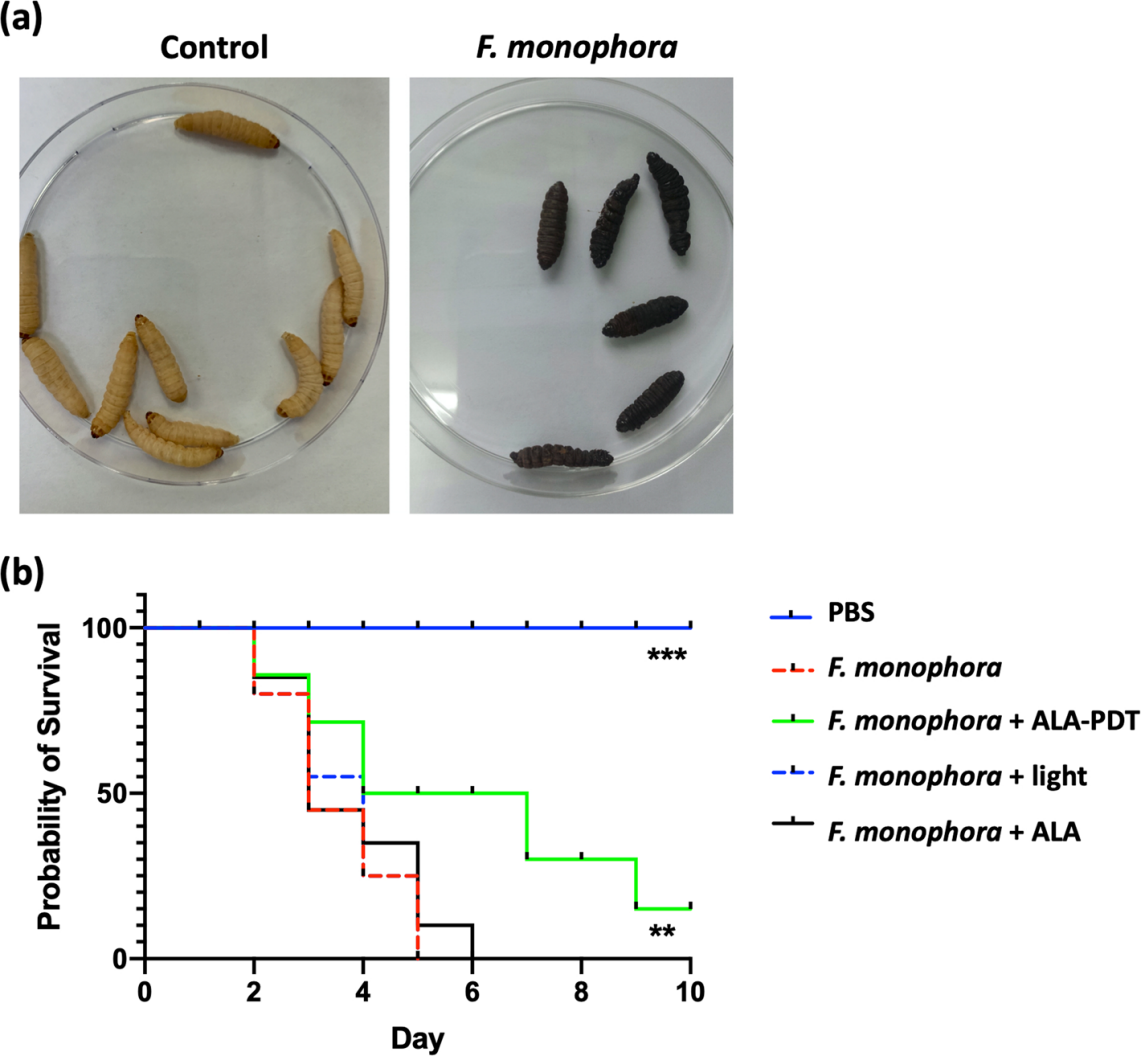
ALA-PDT prolongs the survival of *G. mellonella* infected with *F. monophora.***a** The selected *G. mellonella* before infection (left). After 5 days, larvae became melanization over the infection with *F. monophora* (1 × 10^6^ cells per larva) (right). Representative larvae were picked into Petri dishes and photographed. **b** The survival curve of larvae. *G. mellonella* larvae were injected with *F. monophora* (1 × 10^6^ cells per larva) or PBS as the control. *n* = 15. Treatments were performed 2 h after the infection. ALA-PDT, ALA-mediated PDT treatment. Light, procedure control. ALA, reagent control. The experiment was repeated thrice on different days. The representative results were displayed. **, *p* < 0.01; *** *p* < 0.0001

According to our previous research [8], a lethal concentration of 10^6^ cells/larva was adopted to perform the experimental infection with *F. monophora* in larvae. We monitored the survival of larvae once daily and drew the survival curve. *F. monophora* caused melanization of *G. mellonella* (Fig. 1a) and was lethal to larvae. It killed all the larvae within 6 days at the concentration of 10^6^ cells/larva (Fig. 1b). After confirmed the *G. mellonella* infection model was working, we applied ALA-PDT on larvae 2 h post the infection. Figure 1b showed that with the ALA-PDT application, the survival of infected larvae prolonged significantly compared to the untreated ones. The median survival (time after half of animals have survived) in larvae receiving PDT treatment extended to 5.5 days from 3 days. There was no difference between the untreated group and the groups of receiving light exposure or photosensitizer injection independently.

### Safety evaluation of ALA or MB-medicated PDT in *G. mellonella*

**Fig. 2:**
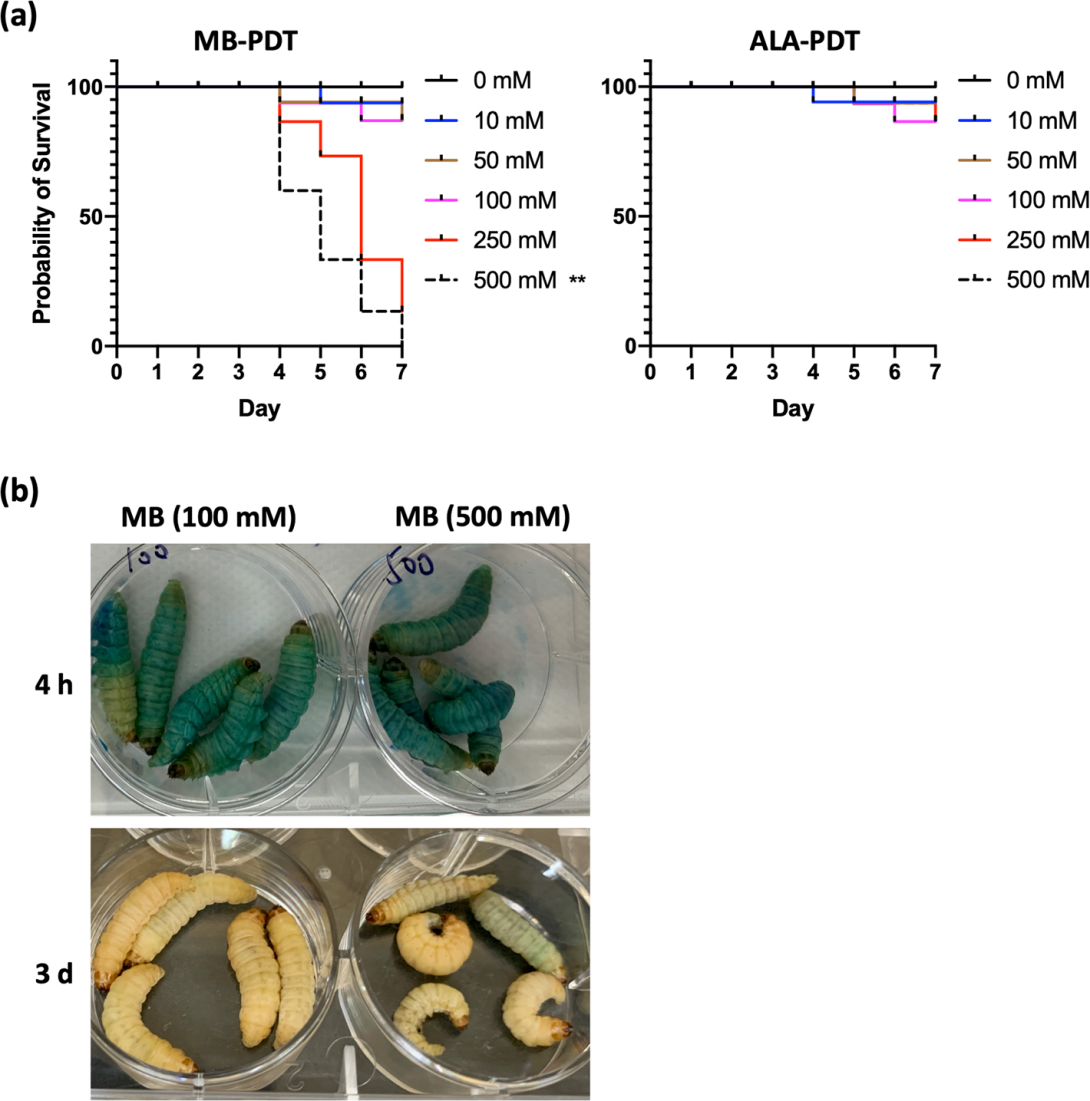
Safety evaluation of ALA or MB-medicated PDT on *G. mellonella.***a** The survival curves of larvae. *G. mellonella* larvae were inoculated with different concentrations of MB or ALA solutions, and observed daily for 7 days. PBS was used as control. n = 15. The experiment was repeated thrice on different days. The representative results were displayed. **, *p* = 0.006. **b** The visualized diffusion of MB within larvae 4 h or 3 d post-injection. Photos were taken for the representative larvae

We next wondered about the safety of PDT when applied to larvae. The toxicological evaluation was performed to verify the process of PDT treatment. As shown in Fig. 2a, we examined the toxicity of ALA and MB, which are the most frequently used photosensitizers for patients. The larvae were treated with photosensitizer (ALA or MB) at different concentrations (from 10 mM to 500 mM) following with light-irradiation. After PDT treatment, the survival curve was depicted with daily observation. No significant difference existed in safety between the ALA-mediated PDT group and the control group with PBS treatment. However, MB-mediated PDT induced significant death of larvae at a high concentration of 500 mM. 40% of larvae injected with MB died from day 4 post the irradiation, and there was no living left after 1 week. Results also showed that all larvae were alive in low concentrations of the MB group and the ALA group during the observation. In addition, it was visible of MB diffusion within larvae during the initial 4 h post-injection. The solution of MB featured in blue. As shown in Fig. 2b, the larvae remained a bit blue on the skin surface after 3 days of MB application, which indicated the diffusion of MB in larvae. The slow diffusion might connect to the toxicity as observed with 500 mM MB (Fig. 2a).

### In vitro effect of ALA-PDT treated larval hemolymph/hemocyte on *S. aureus* and *C. albicans*

**Fig. 3:**
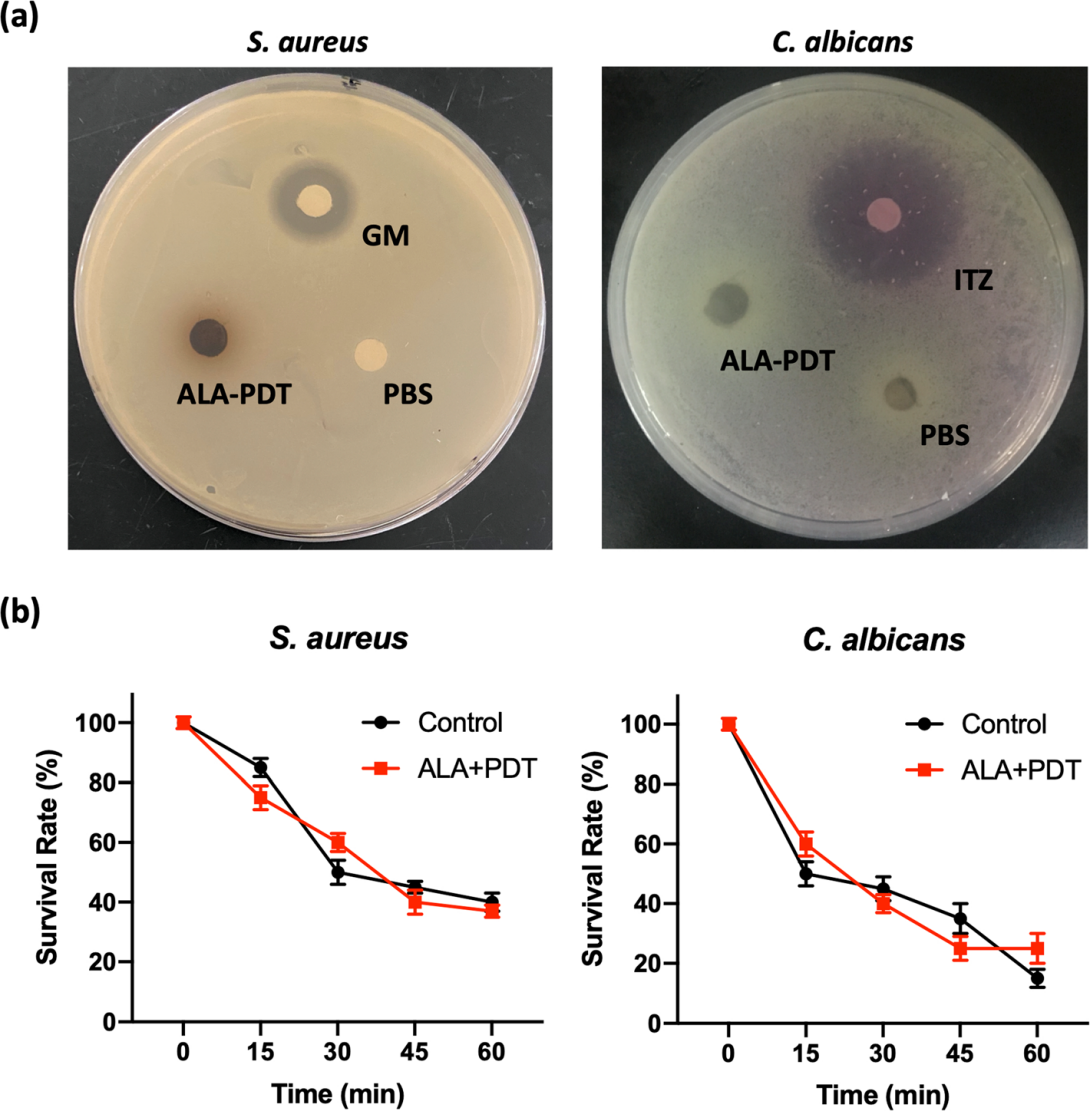
Hemocytes from ALA-PDT treated larvae do not contribute to the killing of *S. aureus* and *C. albicans* in vitro*.***a** The disk diffusion assay against *S. aureus* and *C. albicans*. The larval hemolymph (containing hemocytes) was collected after ALA-mediated PDT treatment. PBS, negative control was the blank paper disk impregnated with PBS. GM or ITZ, positive control was the blank paper disk impregnated with gentamicin (GM, 1 mg/mL) or itraconazole (ITZ, 16 mg/L). **b** Kinetics of bacterial and fungal killing by insect hemocytes. Five larvae were treated with ALA-PDT. PBS was used as control. The hemocytes were collected and pooled. *S. aureus* or *C. albicans* cells were exposed to hemocytes at the indicated timepoints. The data are from a representative experiment performed in triplicate

Further, the inactivation of fungi by larvae receiving ALA-PDT treatment was detected in vitro. As shown in Fig. 3a, the disk-diffusion assay demonstrated that hemolymph collected from ALA-PDT treated larvae were non-inhibitory to *S. aureus* and *C. albicans*, with no obvious diameters of growth inhibition. However, we noticed the melanization and congelation of hemolymph displaying on the disk, which might have some adverse impacts on the diffusion assay.

The killing efficacy of bacteria and fungus by hemocytes was next determined. We monitored for 60 min and found that the killing of *S. aureus* occurred more slowly and weakly compared to that of *C. albicans* group (Fig. 3b). The killing of *C. albicans* was visible from the initial 15 min and achieved a 50% ± 4.5% reduction of viable fungi yeast at 30 min, while 15% ± 3% of *S. aureus* were killed after 15 min. Notably, there was no statistical difference in killing assays between ALA-PDT treated and untreated groups. It indicated that ALA-PDT did not enhance the killing of bacteria or fungi by larval hemocytes.

### Effect of ALA-PDT on hemocyte density and susceptibility

**Fig. 4:**
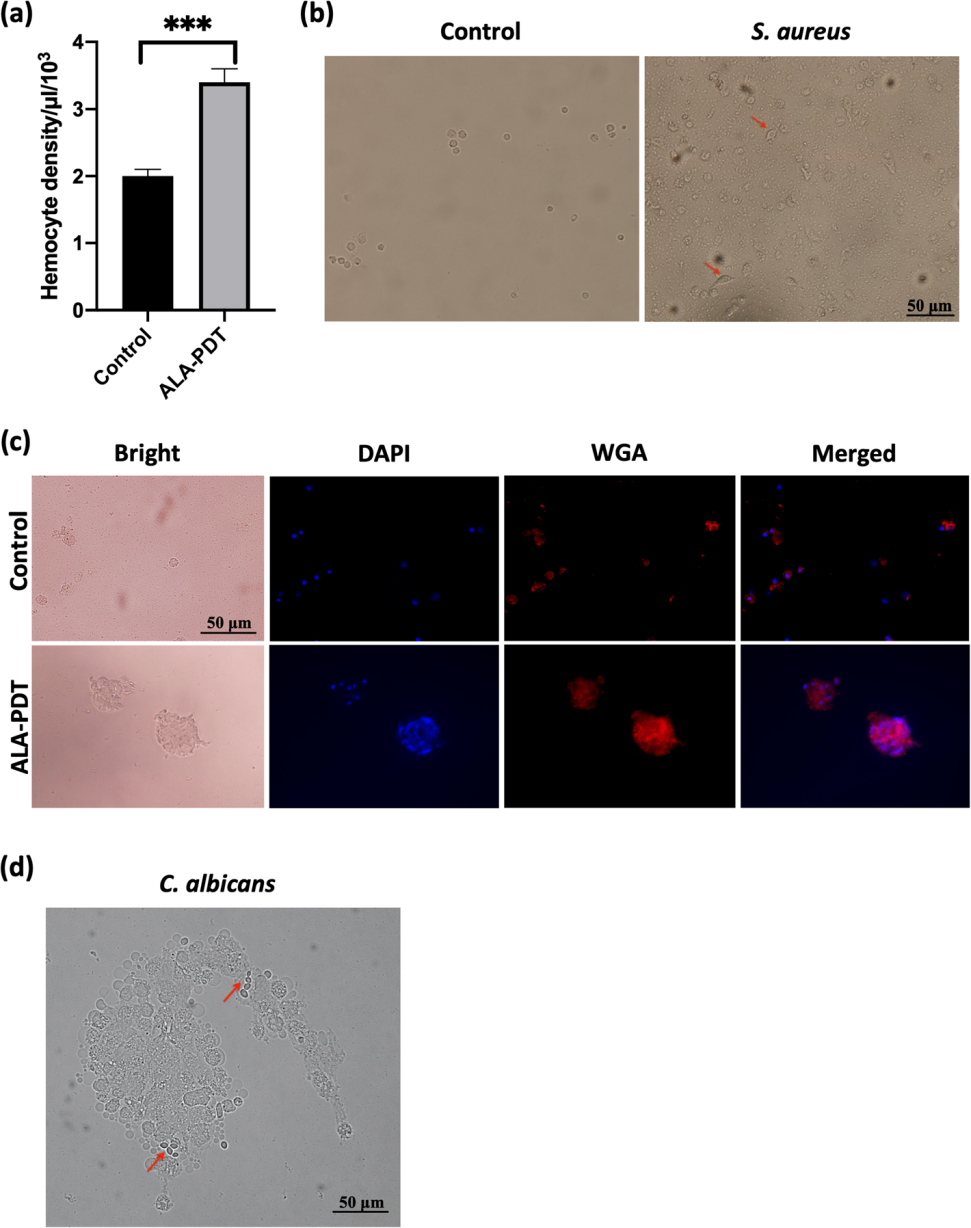
ALA-PDT improves hemocyte density and susceptibility to pathogens. Hemocytes were taken and pooled from five larvae for each experiment group. **a** Quantification of *G. mellonella* hemocytes. ALA-PDT, *G. mellonella* received ALA-mediated PDT treatment. Control, *G. mellonella* were injected with PBS. ***, *p* < 0.001. **b** Hemocytes were stimulated with *S. aureus* (MOI = 2) for 3 h (right). Arrow indicated the morphological changes of hemocyte after stimulation of *S. aureus*. Scale bar, 50 μm. **c** Staining of presentative hemocytes. Larvae were pretreated with ALA-PDT for 4 h. Hemocytes were collected and incubated with *S. aureus* (MOI = 2) for 3 h. DAPI (300 nM) labeled the nuclear of hemocytes and WGA (10 μg/mL) labeled *S. aureus* cell wall. Scale bar, 50 μm. **d** The aggregation of *G. mellonella* hemocytes to conidia of *C. albicans*. Hemocytes collected from *G. mellonella* with receiving ALA-PDT treatment were inoculated with *C. albicans* (MOI = 2) for 2 h. Arrow, *C. albicans* conidia. Scale bar, 50 μm

Since there was no significant improvement in direct-killing of *S. aureus* or *C. albicans* by hemolymph with the treatment of ALA-PDT in vitro (Fig. 3b), we further investigated the ability of ALA-mediated PDT for inducing the immune response in *G. mellonella*. Figure 4a showed the increased presence of hemocytes 4 h after PDT treatment, with the recording of 3.41 ± 0.22 × 10^3^ cells/μL, which differs significantly from hemocyte density of 2.07 ± 0.12 × 10^3^ cells/μL in the untreated larvae (*p* < 0.001). Therefore, ALA-PDT did affect larval hemocyte density.

We continued to measure characteristics of hemocytes under the challenging with *S. aureus* or *C. albicans*. As shown in Fig. 4b, *G. mellonella* mock hemocytes varied in shape after stimulated with *S. aureus*. The cells formed pseudopodia with spindle-shaped cell surfaces, while non-stimulated ones were small round cells with smooth surfaces. Moreover, immunofluorescence was performed to characterize the phagocytosis of *S. aureus* by larval hemocytes. After PDT treatment, in response to *S. aureus*, hemocytes gathered together in large size and were highly adherent to the exogenous bacteria, which was stained with WGA (Fig. 4c). In the challenge of *C. albicans*, pretreatment with PDT induced more aggressive hemocyte agglomeration and pigmented cores. Conidia of *C. albicans* were wrapped up by hemocyte, where the arrow pointed (Fig. 4d). The increasing presence of susceptible hemocytes indicated that ALA-PDT had the ability to enhance immune responses of *G. mellonella*.

## Discussion

Clinical application of PDT in dermatology has been developed recently and focused on skin infection caused by many types of pathogens [11]. Especially, in our clinical practice, we observed that ALA-mediated PDT was working well in removing CBM lesions [2, 3]. Here, we proved that treatment of ALA-mediated PDT, comparing with MB-mediated PDT, is safe for *G. mellonella* larvae. The application of ALA-PDT on *G. mellonella* can help host defense against infection by improving host hemocyte density and susceptibility to pathogens.

Previously, we had used the larvae of *G. mellonella* to construct an experimental infection model [8, 12], which is helpful for the development of pathogenesis research and novel treatments. Meanwhile, *G. mellonella* is a PDT-susceptible organism [13, 14]. This invertebrate is economical and easy for performing experimental procedures, which makes it used as an alternative model to study pathogenesis and virulence factors of diverse bacterial and fungal pathogens [5, 15].

Some researchers found that the antifungal capacities of PDT are positively related to the photosensitizer (MB) concentration within *G. mellonella* [16, 17]. High dose of photosensitizer might achieve better efficacy of treatment. Our data here showed that a lower dose of MB was not harmful to larvae receiving MB-mediated PDT, while a higher concentration of MB could make the host weak and dead. Compared to MB, there was no significant death of larvae with ALA treatment, even at the highest concentration. Therefore, we chose ALA as the photosensitizer in our current study. To our knowledge, this is the first trail to perform a high concentration (500 mM) of photosensitizers in *G. mellonella* for examining the treatment toxicity. In order to observe the antimicrobial effect of larval hemolymph, the disk diffusion assay and in vitro killing assay were performed. It is evidenced that hemocytes had the ability to inhibit the growth of *S. aureus* and *C. albicans*. However, we did not find a statistically significant difference between PDT treated and the untreated larvae. It means ALA-PDT does not contribute to the antimicrobial effect of *G. mellonella*.

It is known that the immune system of *G. mellonella* has structural and functional similarities to the innate immune response of mammals [9]. Due to the similarity, larvae have been increasingly used in antimicrobial investigations. The insect hemolymph contains circulating hemocytes with the capacity to immobilize and/or kill microorganisms [18]. Dos Santos et al. reported PDT activates the *G. mellonella* immune system by increasing circulating hemocytes against *Porphyromonas gingivalis* infection [19]. In the present study, we observed the application of ALA-PDT on larvae infected with *F. monophora* prolonged the survival of larvae. According to Chibebe et al. [20], PDT facilitates the insect immune system response to solve the infection, consistent with our results. Moreover, we further demonstrate in detail that ALA-mediated PDT could increase hemocyte density and susceptibility to pathogens. As the dominant player in *G. mellonella* innate immunity, hemocytes with boosted function by PDT could effectively eliminate the invading pathogen. This study confirmed that PDT has immunomodulatory effects in the *G. mellonella* model animal.

## Conclusions

In this study, we investigated the safety for the administration of PDT in model animal *G. mellonella* with two different photosensitizers (ALA and MB) at serial concentrations (10 mM to 500 mM). ALA-mediated PDT displayed non-toxic compared with MB-mediated PDT. Indeed, ALA-mediated PDT could save *G. mellonella* from fungal infection of *F. monophora*. Since that ALA-PDT could increase hemocyte density and susceptibility to pathogens, investigations of *G. mellonella* infection models is beneficial for understanding PDT-mediated immunoregulatory effect. Overall, this study implies that PDT implementation in *G. mellonella* facilitates a detailed understanding of the antimicrobial property and immunomodulation of PDT.

## Methods

### Strains of microorganism

A clinical isolate of *F. monophora* was recovered at Sun Yat-sen Memorial Hospital, Guangzhou, China, and the identification was carried out as described previously [8]. In order to induce the formation of conidia, the *F. monophora* isolate was subcultured from Sabouraud dextrose agar to potato dextrose agar (Sigma-Aldrich, St. Louis, MO, USA), and kept at 26 °C for 10 days. Standard strains of *C. albicans* (ATCC 18804) and *S. aureus* (ATCC 12600) were purchased from American Type Culture Collection (ATCC, Manassas, VA, USA), and stored in frozen stocks with BHI broth (Sigma-Aldrich, St. Louis, MO, USA), containing 20% glycerol at − 80 °C. *S. aureus* was grown on LB plates (Sigma-Aldrich, St. Louis, MO, USA) at 37 °C for 24 h before the use.

### *G. mellonella* infection models and the killing assay

The *G. mellonella* infection was carried out as described previously [12]. Briefly, *G. mellonella* in the final instar larval stage (300–350 mg in body weight) were selected and distributed randomly into different groups. Every infection set consisted of 5 larvae. Triplicate sets of 15 larvae were employed for every condition in all killing assays. Ten μL of the conidia inoculum with 1 × 10^6^ cells were injected into the hemocoel of the larvae via the last left proleg. The same volume of PBS (Thermo Scientific, Wilmington, DE, USA) was injected as the control. After inoculation, the infected larvae were placed in disposable Petri dishes and incubated at 37 °C. The number of dead larvae was scored daily for the survival curve. The larvae were considered dead when they displayed no movement in response to touch and removed from the dishes.

### Application of photodynamic therapy on *G. mellonella*

The fresh solution of photosensitizers MB and ALA (Sigma-Aldrich, St. Louis, MO, USA) were prepared for each experiment. A volume of 10 μL of the photosensitizer solution at different concentrations (from 10 mM to 500 mM) was injected into the larvae via the last right proleg. Every treatment set consisted of five larvae. Triplicate sets of 15 larvae were performed. Then the larvae were maintained in the dark for 30 min as the pre-irradiation time. The laser light (PDT, LED-IB, Wuhan Yage, China) was applied to larvae contained in the 6-well plate for 25 min. After irradiation, larvae were transferred to the dark incubator and kept at 37 °C for the required time. For larvae infected with *F. monophora*, the application of PDT on larvae was performed 2 h after inoculation with fungal spores.

### Quantification of *G. mellonella* hemocytes

A volume of 10 μL of photosensitizer ALA at the concentration of 100 mM was used for PDT treatment on *G. mellonella*. The larvae were exposed to red light 30 min after the injection of ALA. Then, the treated larvae were incubated at 37 °C for 4 h, and hemolymph was extracted. Every treatment set consisted of six larvae and repeated trice. The hemolymph was collected into 1.5 mL Eppendorf Safe-Lock Microcentrifuge Tube (Sigma-Aldrich, St. Louis, MO, USA) containing ice-cold Grace’s medium (Thermo Scientific, Wilmington, DE, USA). Then hemocytes were purified after centrifugation and quantified by the hemocytometer.

### The disk diffusion assay

Isolate of *C. albicans* and *S. aureus* was respectively grown in YPD and LB liquid medium (Sigma-Aldrich, St. Louis, MO, USA) overnight. The next day about 1 × 10^5^ yeast and bacterial cells were spread on a rich medium plate (10 cm Petri dish), separately [21]. Hemolymph was extracted from larvae 4 h post-PDT and diluted with PBS in the ratio of 1:1. Every treatment set consisted of five larvae. Triplicate sets of 15 larvae were performed. The blank round paper disks (eight mm in diameter) were impregnated with prepared hemolymph or PBS control. Then, prepared disks were placed triangularly on *C. albicans* and *S. aureus* plates, and the plates were incubated in the dark at 35 °C for 24 h. Then photographed the plates and compared the diameters of the inhibition zones. Each assay was performed thrice.

### The in vitro bacterial and fungal killing assay

Fifteen larvae per group with or without PDT treatment were used for colony-forming units (CFU) counting. The hemolymph of larvae was collected 4 h post light exposure for killing assay. Hemocytes (1 × 10^7^) were incubated with *S. aureus* (2 × 10^7^) or *C. albicans* (2 × 10^7^) in a chamber at 37 °C. The killing of *S. aureus* and *C. albicans* were measured every 15 min. The survival observation was performed through CFU determination. The survival rate was calculated by CFU at every time point comparing with the initial bacterial load.

### Microscopy

Hemolymph was extracted from *G. mellonella* 4 h post-ALA-PDT and collected for purifying hemocytes. Bacterial cells of *S. aureus* were stained with Wheat Germ Agglutinin (WGA) labeled with Biotium’s Dye CF555 (Biotium, Inc., Hayward, CA, USA) at 37 °C in the dark for 15 min, and co-cultured with hemocytes at the ratio of 2:1 in the 96-well plate for 3 h. After washing with PBS thrice, hemocytes were stained with DAPI (Thermo Scientific, Wilmington, DE, USA) in the dark for 10 min, then mounted on glass coverslips and viewed directly using an Olympus BX63 fluorescence microscope.

### Statistical analysis

Data were analyzed by using the SPSS software 15.0 (SPSS Inc., Chicago, IL, USA). *G. mellonella* survival was examined using the Kaplan-Meier method, and differences were determined by using the log-rank (Mantel Cox) test. Difference in hemocytes density and bacterial/fungal killing assays was assessed by ANOVA analysis followed by the Tukey’s test. A *p*-value of 0.05 in all replicate experiments was considered statistically significant.

## Data Availability

All data generated or analyzed during this study are included in this published article. Access to raw data can be acquired by connecting to the corresponding author via email.

## Abbreviations

ALA: 5-aminolevulinic acid
*C. albicans*: *Candida albicans*
CBM: Chromoblastomycosis
*F. monophora*: *Fonsecaea monophora*
*G. mellonella*: *Galleria mellonella*
MB: Methylene blue
PDT: Photodynamic therapy
*S. aureus*: *Staphylococcus aureus*
